# A new multidimensional group intervention for cognitive and psychosocial functioning for older adults: Background, content, and process evaluation

**DOI:** 10.3389/fmed.2023.1161060

**Published:** 2023-04-20

**Authors:** Andreas Chadjikyprianou, Fofi Constantinidou

**Affiliations:** Department of Psychology and Center for Applied Neuroscience, University of Cyprus, Nicosia, Cyprus

**Keywords:** intervention, aging, cognitive aging, psychosocial functioning, cognitive functioning, emotion regulation, memory compensation strategies, locus of control

## Abstract

**Introduction:**

An essential element of quality aging is the maintenance of cognitive and psychosocial functioning. The principal objective of the present paper was to present the theoretical framework, content and process evaluation of a newly developed multi-dimensional group intervention designed to strengthen/improve areas of cognitive and psychosocial functioning in adults over 65.

**Methods:**

The intervention implements multiple methodologies aiming to facilitate contextual integration of learned concepts and strategies derived from clinical psychology and rehabilitation. It moves seamlessly on the cognition–emotion axes and consists of five active ingredients selected to address challenges associated with aging: Memory Compensatory Strategies, Problem-Solving, Emotion Regulation, Mindfulness, and Locus of Control. Thirty participants joined the intervention group aged 65–75  years (*M* = 69.03; SD = 3.04). All 30 participants who were included in the intervention group completed the program.

**Results:**

Results from the Participant Satisfaction Scale indicate that the program was perceived very positively by participants, who also reported implementing their newly learned strategies in activities of daily life. Furthermore, there was high correlation between internal locus of control and the learned strategies.

**Discussion:**

The outcomes of this analysis indicate that the intervention is feasible and well tolerated by our target group. This multidimensional intervention may offer a valuable contribution to public health care and dementia prevention for older adults.

**Clinical Trial Registration:**

[https://clinicaltrials.gov/ct2/results?cond=NCT01481246], identifier [NCT01481246].

## Introduction

The increase in life expectancy and health concerns associated with demographic aging is of global interest. An essential element of quality aging is the maintenance of brain health and actions to prevent dementia (1).

The literature supports a biopsychosocial perspective aiming at the development and implementation of interventions that target concepts such as successful aging, active aging and healthy cognitive aging (2, 3). Common to all concepts, is the need to develop and maintain functional capacity of older people, emphasizing in psychosocial and cognitive factors.

In line with the biopsychosocial approach, there has been a growing interest in interventions that adopt a multidimensional approach to cognitive aging (4). Some studies used a multidimensional approach for raising awareness of modifiable factors that contribute to cognitive decline such as physical activity, nutrition, interpersonal relations, and stress management, showing a significant increase in raising awareness after the intervention, indicating the effectiveness of such educational programs (5, 6). Another study used everyday memory and metacognitive intervention to improve everyday functioning (7) by focusing on improving older adults’ ability to achieve cognitively challenging everyday life tasks. Results showed improvements in everyday memory functioning and extended functional independence. Hoogenhout et al. (8), focused on psychoeducation for cognitive health through the use of self-regulation over habitual behaviors that often interfere with daily functioning. Their findings indicated reductions in negative emotional reactions toward cognitive aging, which, they assert is a prerequisite for improved subjective cognitive functioning and well-being. Finally, Ruvalcaba and Merino (9) promoted quality of life, physical activity, improved nutrition and cognitive function, through the use of theoretical–practical intervention. Results showed improvements in self-efficacy for quality of life, physical activity, nutrition and cognitive function.

The present research is part of a systematic effort to design and implement interventions that promote healthy cognitive aging, targeting cognitive and psychosocial abilities. The new intervention extends previous research and integrates knowledge from clinical psychology and cognitive rehabilitation. It conceptualizes five active ingredients which are trained sequentially and in parallel and targets multiple outcomes. Furthermore, the intervention goes beyond the simple didactic mode of transmitting information, to the implementation of role-play and case scenarios from real life to provide opportunities for application of the new skills in the real-life context. At the same time, the intervention addresses a relatively stable component of personality, that of locus of control, whose modification is targeted through the intervention. The purpose of the present article, is to describe the intervention’s theoretical background, content and methodology in order to enable application and replication by researchers in cognitive and psychosocial rehabilitation. In addition, it provides information on the intervention’s feasibility and acceptability by the participants as recommended by the literature (8, 10, 11).

### Integrating cognitive and psychosocial dimensions for the group intervention

The present novel multidimensional, non-pharmaceutical group intervention integrates key theoretical principles from clinical psychology and cognitive rehabilitation to improve cognitive and psychosocial functioning in adults over 65. The intervention moves seamlessly on the cognition–emotion axes and consists of five active ingredients selected to address challenges associated with aging: Memory Compensatory Strategies, Problem-Solving, Emotion Regulation, Mindfulness, and Locus of Control. These active ingredients were specifically selected as they are important for the cognitive and psychosocial well-being of older people and deficiencies in these areas may pose a threat to independent living and successful aging. The intervention integrated concepts of cognitive rehabilitation such as training in memory compensation strategies and problem-solving and from clinical psychology such as applied emotional regulation strategies, mindfulness, and locus of control, bringing to light the “hot” emotional aspects of executive functions (12). Hot executive functions include future-oriented cognitive processes relevant to environments or situations that can generate emotions and encompass motivation, decision making and social behaviors (13). Those connections between emotion and cognition help maximize functionality in daily life.

The above elements are important novel aspects of this intervention. As an example, previous interventions make reference to stress management through relaxation techniques (5) or the reduction of negative emotional reactions through positive thinking (14). The present intervention, in addition to relaxation to assist with stress management, incorporates concepts of mindfulness and emotion regulation in conjunction with problem-solving strategies, to build connections between emotion and cognition and to help maximize functionality in daily life.

**Figure 1:**
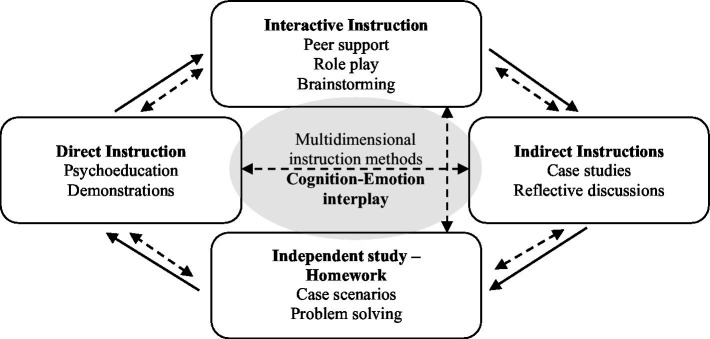
Multidimensional instruction methods.

**Figure 2:**
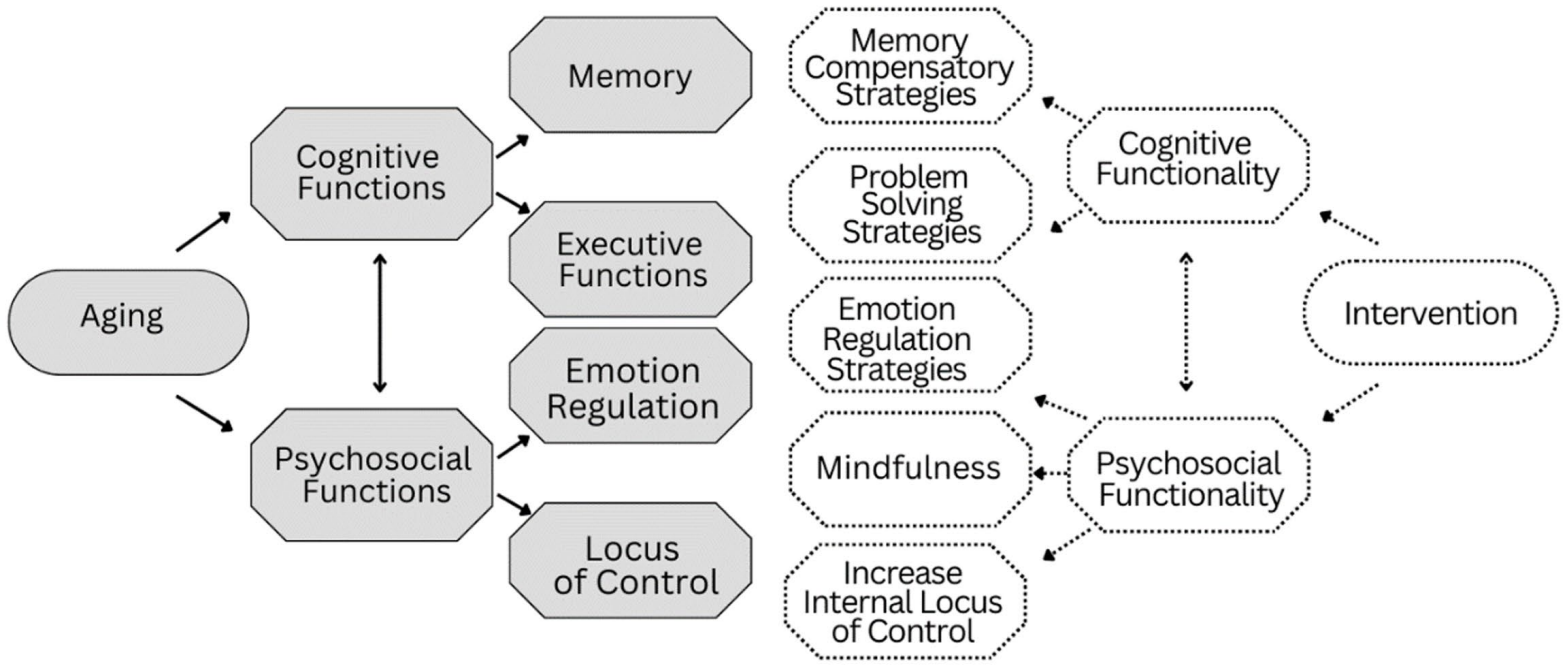
Conceptual framework of the intervention program.

Participants were trained on the interplay between cognition and emotion and the 10-week group intervention incorporated didactic (psychoeducational) methodologies, role-play, peer support, and take-home exercises for contextual integration of learned concepts and strategies. Figure 1 presents the multidimensional instruction methods used. Each session begins with direct instruction, which continues with interactive instruction and indirect instruction and concludes with independent study. In all methods, both cognitive and emotional processes are trained. During the program, the various teaching methods are aligned to positively reinforce each other. For example, during training in the use of Memory Compensation Strategies, interactive teaching methods such as role play and peer support, reinforce indirect teaching such as case studies, which in turn facilitate the participant’s ability to complete homework and in turn reinforce indirect teaching methods such reflective discussions in the next session. Figure 2 presents the conceptual framework of the design of the intervention. The following sections provide a description of the key active ingredients and the primary outcome measures associated with each ingredient.

### Memory compensation strategies

Since memory problems are the most obvious complaints in older people (15), most interventions have focused on strengthening the mnemonic function and the implementation of compensatory strategies. Memory Compensation Strategies (MCS) refer to a group of mechanisms through which individuals can continue to perform well in complex tasks despite having experienced deficits or reductions in memory capacity (16). MCS were incorporated as a key cognitive strategy aiming to train participants to develop effective strategies that support effective information coding (17). Participants were trained in the use of eight internal (e.g., semantic correlation and repetition) and external strategies (e.g., notes and placing reminders) derived from educational psychology (18), and cognitive rehabilitation (19, 20). Importantly, the new intervention encouraged participants through in-house and takes home exercises to learn and incorporate new MCS strategies in addition to their established ones. Improvement in this area was measured by “Scale of Implementation of Memory Compensation Strategies (21).”

### Problem solving strategies

Along with MCS, Problem-solving (PS) training is an important aspect of cognitive interventions designed to enhance cognitive and psychosocial functioning (19, 22). PS is considered a component of executive functioning, a cognitive domain which, like memory, is also vulnerable to the effects of healthy aging (23–25). Older adults without neurodegenerative disease or mild cognitive decline, may demonstrate difficulties in problem solving and decision making (22, 26, 27).

The present study presented and trained participants in a five-step PS process as part of the “Think & Act” section. The PS training included four phases: (a) Brainstorming: participants were guided in generating possible and alternative solutions to solve a problem; (b) Planning and organization: individuals were guided in evaluating and comparing the solutions created and in defining the analytical implementation strategy for the selected solution; (c) Role play: practicing the strategies to scenarios presented by the researcher during the session; and (d) Generalization: applying their newly learned skills to a real-life problem at home as part of their homework assignment.

As part of the PS training, participants were guided in identifying areas of possible improvement for their overall health. Lifestyle factors such as nutrition and physical activities/movement were targeted as overall strategies promoting physical health and well-being. Specifically, deficiencies in those areas were identified individually for each participant and were approached with the PS process in order to implement positive changes to overall health. Improvement in this area was measured by “Problem-Solving Inventory” (28, 29).

### Emotional regulation strategies

Emotional regulation (ER) is an important element of effective problem solving (30) and a key dimension of psychosocial functioning. ER is also considered an indicator of positive adaptation to the negative aspects of aging (31). ER was included in this study as a ‘hot’ area of executive functioning (32, 33) with the aim of enhancing psychosocial and cognitive functionality. ER was addressed through the “Stop & Relax” process which incorporated key elements of regulating emotions as described by Gratz and Roemer (34). Participants were trained to recognize, name and accept emotions but also to use diaphragmatic breathing in order to relax, reduce physical arousal and implement effective PS strategies using the “Think & Act” techniques. They were asked to maintain a weekly diary of emotional clarity with the aim of identifying and accepting emotions as well as practicing diaphragmatic breathing weekly. Improvement in this area was measured by “Difficulties in Emotion Regulation Scale” (34, 35).

## Mindfulness

Mindfulness refers to the present-oriented attention within the self (e.g., body sense, thoughts, feelings) and outside (environment) without criticism (36). Research on mindfulness indicates that it is a beneficial modality for ER, EF, and memory (37–40). However, research on mindfulness and aging is understudied. The present study incorporated mindfulness as part of ER and the “Stop & Relax” procedures to facilitate emotional functioning, problem-solving, executive functioning, and memory performance. The combination of ER and mindfulness is thought to have an additive effect as compared to using each of the two in isolation. The implementation of mindfulness and other strategies addressed in the program, aimed at improving locus of control. Improvement in this area was measured by “Mindful Attention Awareness Scale” (41, 42).

### Internal locus of control

Locus of Control (LC) is a psychological construct that evaluates beliefs about one’s ability to influence situations in their lives (internal LC) or whether these are left to external forces (external LC) (43). Research suggests that biological aging interferes with the sense of control in older people (44, 45). This decrease in perceptual control is clearly related to the increasing diffusion of age-related barriers and restrictions, such as biological and social changes (e.g., retirement, reduced incomes, cognitive changes, bereavement) (46).

LC may be modifiable in response to both life events and after training (46, 47). The present intervention integrates LC in all aspects of the program by empowering participants to gain knowledge the various aspects of aging, on implementing strategies for healthy physical and cognitive aging and applying methods to promote memory, PS and ER. Participants were encouraged to discuss personal obstacles and were guided to take systematic guidance actions in order to improve life outcomes and reduce beliefs that their lives depend on external factors (luck, strong others). The shift from external LC to internal LC could facilitate the implementation of cognitive and emotional strategies to promote healthy cognitive aging. Improvement in this area was measured by “Multidimensional Locus of Control” (29).

The aim of the present study was the implementation of a multi-dimensional group intervention designed to strengthen areas of cognitive and psychosocial functioning in adults over 65. The intervention implements multiple methodologies aiming to facilitate contextual integration of learned concepts and strategies derived from clinical psychology and rehabilitation. The intervention is novel as it moves seamlessly on the cognition–emotion axes and consists of five active ingredients selected to address challenges associated with aging: Memory Compensatory Strategies, Problem-Solving, Emotion Regulation, Mindfulness, and Locus of Control. It was hypothesized that participants would perceive the intervention as a useful approach to improve cognitive and emotional functioning and that those who demonstrated high levels of internal locus of control would report greater engagement in implementing their new knowledge in activities of daily living.

## Methods

### Participants

Sixty-nine community dwellers were initially recruited to participate in this study. Nine people were excluded due to non-compliance with the admission and exclusion criteria. This research was incorporated into the Neurocognitive Study of Aging (NEUROAGE) which is the first systematic and longitudinal study of cognitive aging in Cyprus (NCT01481246) (24, 48).

**Figure 3:**
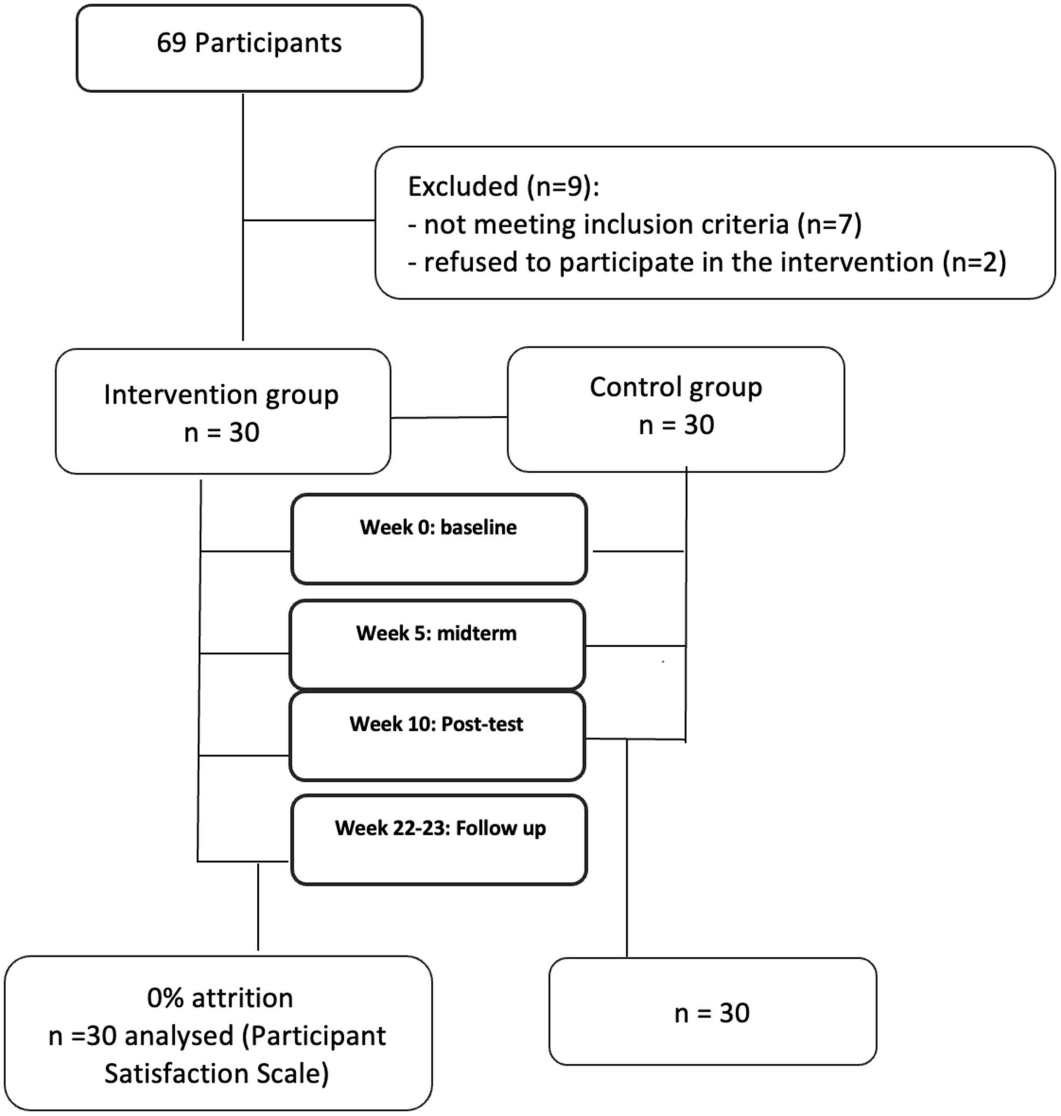
CONSORT flowchart.

The remaining 60 participants were randomly assigned into the intervention and control groups. Thirty people joined the intervention group, of which 19 were women and 11 men aged 65–75 years (*M* = 69.03; SD = 3.04). The control group also consisted of 30 people, 21 of whom were women and nine men, aged 65–75 (*M* = 70.12; TA = 3.51). The level of education was quantified as the number of years of formal education achieved by each participant and ranged from 8 to 17 years (*M* = 11.7, SD = 2.11) for the intervention group, and for the control group (*M* = 11.76, TA = 2.02). The two groups were matched on key demographic variables as well as on the Mini-Mental State Examination performance (*M* = 28.31, SD = 1.80 for the intervention group and *M* = 28.06, SD = 1.95 for the control group). Figure 3 is the CONSORT diagram.

The inclusion criteria for all participants were the following: (1) Native Greek speakers; (2) males and females over the age of 60; (3) Good general health with no previous history of neurological disorder such as head trauma, stroke or neurodegenerative disorder; (4) No emotional or psychiatric disorder as defined by the Diagnostic-Statistical Manual of Mental Disorders, Fifth Edition (DSM-V); (5) Results on neurocognitive tasks falling within the normal range as derived from normative data of the NEUROAGE (within 1 and − 1 *z* scores).

### Measures

Participants were initially evaluated with the neurocognitive evaluation lasting about 2 hours, which examined the general mental function, memory, executive functions and language as described by our previous work (24, 48). This evaluation was conducted to ensure normal cognitive function for the study participations.

In addition to the neurocognitive evaluation, the study implemented the following primary outcome (dependent) measures relevant to the theoretical components of the intervention and were administered at baseline, posttest and follow-up:

1. Multidimensional Locus of Control Inventory (29, 49).
2. Problem-Solving Inventory (28, 29).
3. Difficulties in Emotion Regulation Scale (34, 35).
4. Scale of Implementation of Memory Compensation Strategies (21).
5. Mindful Attention Awareness Scale (41, 42).

### Participant satisfaction scale

For the purpose of this intervention, a questionnaire consisting of 18 statements was created to investigate the satisfaction of participants in key areas of the intervention and their ability to apply the new knowledge and skills in their daily lives. The questionnaire consists of three parts. The first part consists of statements in which participants rate their satisfaction with general aspects of the intervention such as the topics addressed in the meetings, the organization and preparation of the instructor and satisfaction with the instructor. Questions could be answered on a 5-point scale (1 = very dissatisfied, 5 = very satisfied). The second part consists of statements assessing the degree of agreement on issues specific to the intervention such as gaining knowledge on healthy cognitive aging, on overall motivation to increase locus of control, and the effectiveness of the group format. Questions could be answered on a 5-point scale (1 = disagrees absolutely, 5 = agrees absolutely). The third part includes statements to evaluate their ability to apply strategies learned during the program in their daily lives (i.e., memory, problem-solving, and Emotion regulation strategies). Questions could be answered on a 5-point scale (1 = poor, 5 = very good). A high internal consistency of the questionnaire was identified in the sample, with Cronbach’s a determined 0.91. Participant Satisfaction Scale was administered at the end of the intervention (10 week).

### Multidimensional locus of control inventory

The Greek version of the Multidimensional Locus of Control was used (29). The scale consists of 24 items scored on a six-point Likert scale, ranging from −3 (Strongly Disagree) to +3 (Strongly Agree). The scale yields three distinct factors: Internality, Powerful Others, and Chance. Each subscale produces a unique score by adding up the eight responses and adding to the sum a constant of +24 to eliminate negative sums. Therefore, each respondent received three scores (each one ranging from 0 to 48) indicative of his/her relative view on each of the three dimensions. An individual could score high or low on all three dimensions. Acceptable internal consistency of the questionnaire was identified in the present sample, with Cronbach’s a determined 0.84 for Internality, 0.79 for Powerful Others, and 0.83 for Chance.

### Study design

Participants were assigned into five groups of 6 people. The intervention was conducted weekly over ten, 90-min sessions, with a mid-point evaluation (at five weeks) and a post-treatment assessment at the end of the intervention. There was a follow-up assessment on the dependent measures 12 weeks after the end of the intervention. To reduce response bias error, all participants completed the questionnaire anonymously and privately; they placed the completed questionnaire in an envelope containing a unique code that was then used to manage the data.

### Intervention procedures

The intervention was designed as 90-min weekly group meetings lasting 10 weeks. The intervention was developed by a doctoral-level licensed and certified academic speech-language pathologist with expertise in cognitive-communication disorders (F.C.) and a doctoral student in Clinical Psychology (A.C.) also licensed in School Psychology, who facilitated all sessions. Another graduate-level researcher monitored the process in each session with the aim of confirming that all groups and meetings followed the same content and procedures. The meetings were originally planned to be conducted in person. However, due to the restrictions implemented to control the spread of COVID-19, the intervention was transformed into an online format. Study participants were trained in person in the use of the Rakuten Viber online platform. This specific platform was selected as participants were more familiar with it and several were using it already in their daily lives. It has numerous options; e.g. it has high-quality video call, better voice quality with less noise throughout all bandwidths, in comparison with other free platforms and provides group chat rooms (50). These elements make this platform suitable for teleconferencing purposes. Viber also provides official desktop version for the apps that can be used in laptops or personal computers. In addition, a family member familiar with the specific platform was available to offer technical support at home during the start of each session. Participants received printed packets of materials required for the program in advance of each group session.

### Presentation format

Each session began with a presentation, followed by direct practice in the use of the proposed strategies through scenarios provided by the researchers as well as through scenarios derived from their personal experience. The content was organized hierarchically aiming to connect the theoretical principles for memory, problem-solving, emotion regulation, and locus of control through psychoeducation and contextual training *via* role play, repetition, and weekly homework with real-life scenarios. The intervention was adaptive in that, e.g., success in week 8 was partly dependent on mastering content from week 2. The intervention’s content is listed in Table 1.

**Table 1:** The main axes of the meetings of the intervention program.

**Week 1**	
Description	Introductions, group procedures, group rules and etiquette, setting goals and expectations
Homework	/
**Week 2**	
Description	Psychoeducation for cognitive aging, development of healthy lifestyle practices/protective factors (such as diet, physical exercise, socialization, involvement with activities that stimulate the brain). Discussion and sharing by group members about their experiences regarding cognitive difficulties/changes Introduction of locus of control and strategies to increase internal locus of control
Homework	Writing goals and monitoring progress in a diary in order to successfully implement actions (areas: diet, physical exercise)
**Week 3**	
Description	Psychoeducation on the memory system and the expected changes associated with increasing age. Training of external mnemonic compensatory strategies
Homework	Calendar of weekly recording of external strategies
**Week 4**	
Description	Training in the use of internal mnemonic strategies. Synthesis of external and internal strategies through everyday scenarios
Homework	Diary of recording external and internal mnemonic strategies. Solving two scenarios with everyday challenges that require the implementation of internal and external strategies.
**Week 5**	
Description	Psychoeducation about emotions and connection with cognitive functions. Discussion on the effects of stress and emotional difficulties on cognitive performance. Developing emotional adjustment skills using the STOP-RELAX approach.
Homework	Logbook of recording external and internal mnemonic strategies. Weekly calendar of recording emotional clarity
**Week 6**	
Description	Further development of emotional regulation skills (diaphragmatic breathing and mindfulness) and gaining knowledge on their effect on the cognitive system. Mindfulness breathing session (5 min)
Homework	Recording of mindfulness goals in the diary. Daily mindfulness breathing and relaxation session. Daily journaling on personal emotional clarity state
**Week 7**	
Description	Psychoeducation on executive functions and association with problem solving. Role of emotions and emotion regulation in problem solving. Five-step technique for problem solving – THINK-ACT, + emotional regulation strategies – STOP – RELAX,
Homework	Solving two scenarios with everyday challenges that requires the implementation of the problem-solving model provided by the instructor. Also, each participant is requested to identify a personal problem and implement the problem-solving model. Continuing the mindfulness training using the audio exercise of mindfulness and diaphragmatic breathing. Daily journaling on personal emotional awareness and clarity state.
**Week 8**	
Description	Deepening in problem solving, the role of emotions and the overall effect on cognitive function. Four strategies of emotional regulation – STOP-RELAX, Five-step technique for problem solving-THINK-ACT,
Homework	The aim this week is to make connections between the newly learned skills with the protective factors for healthy cognitive aging (meeting 2). Participants identified a personal challenge and developed a solution based on the problem-solving strategy. Participants kept a log of external and internal mnemonic compensatory strategies they implemented during daily activities. Daily journaling on personal emotional awareness and clarity state.
**Week 9**	
Description	Synthesis of all previous meetings focusing on problem solving: Protective factors for aging (meeting 2), mnemonic compensatory strategies (meeting 3 and 4), emotional regulation strategies (meeting 5 and 6), problem-solving strategies (meeting 7 and 8)
Homework	In relation to the protective factors for healthy cognitive aging (meeting 2) identification of a different personal goal and its approach based on the problem-solving steps. Diary of recording external and internal mnemonic strategies during activities of daily life. Recording of mindfulness goals and their implementation in the diary. Continuing with the audio exercise on mindfulness and diaphragmatic breathing.
**Week 10**	
Description	Summary, debriefing, feedback by participants, recommendations, closure and planning of reassessment at four weeks and 12 weeks post training.
Homework	Completion of post-testing questionnaires

### Statistical analyses

To investigate the satisfaction of participants in key areas of the intervention and their ability to apply the new knowledge and skills in their daily lives, standard descriptive statistics were utilized, including means, standard deviations and percentages. Pre and post-testing was conducted for all the primary outcome measures and mid-point assessment was conducted for the locus of control and memory strategies. For the purpose of the present study which was to explore potential associations between locus of control (LC) and the self-assessment of participants’ ability to apply their newly trained strategies upon completion of the intervention (at post-test), the post test measures were analyzed. Pearson’s correlations coefficients were performed to examine the relationships. All analyses were carried out with SPSS (version 27).

## Results

### Attrition rate

All 30 participants who were included in the intervention group completed the program. Most of them (94%) attended all 10 sessions. Three participants missed one session and one participant missed two sessions due to illness or family obligations.

### Responses to the participant satisfaction scale

Participant responses regarding the general aspects of the intervention indicate that the majority of participants (90%) were extremely satisfied with the meeting topics (*M* = 4.90, SD = 0.30), with the organization and preparation of the instructor (97%, *M* = 4.96, SD = 0.30), and support provided by the instructor (96.7% *M* = 4.96, SD = 0.18). A high percentage of participants (86.7%) strongly agreed that the meetings were a positive experience (*M* = 4.88, SD = 0.34).

Participants reported high levels of satisfaction for gaining new knowledge and skills. Specifically, 87% indicated that the meetings met their expectations for receiving information related to cognitive health (*M* = 4.50, SD = 0.57) and 90% reported that the meetings met their expectations in developing new skills (*M* = 4.90, SD = 0.30). Specifically, 86.7% reported that they gained knowledge about risk factors for pathological aging (*M* = 4.86, SD = 0.34) and 86.7% that they learned applied strategies they can use in everyday life (*M* = 4.83, SD = 0.42). Most of the participants (76.3%) reported that the meetings motivated them to take control of their life and 76.7% felt motivated to take action for their cognitive health (*M* = 4.76, SD = 0.43). Finally, 63.3% indicated that they applied the general strategies they learned during the intervention for good cognitive health (e.g., healthy diet and exercise) (*M* = 4.56, SD = 0.42).

Regarding the group format of the intervention, 83.3% of participants strongly agreed that this format was optimal (*M* = 4.80, SD = 0.48) and the majority (66.7%) felt a strong connection with their group peers (*MM* = 4.66, SD = 0.59), and 63.7% felt supported by them (*M* = 4.30, SD = 0.66). In Table 2, the means, standard deviations, and percentages for all questions are presented in detail.

**Table 2:** Means, standard deviations, and percentages for the participant satisfaction scale.

	Mean	SD	A and B %	C %	D %	E %
Topics covered during the meetings	4.90	0.34			10%	90%
Organization and preparation of the instructor	4.96	0.19			3%	97%
I felt supported by the instructor	4.96	0.18			3.3%	96.7%
The meetings were a positive experience	4.88	0.34			13.3%	86.7%
The meetings met my expectations for receiving information related to cognitive health	4.50	0.57			3%	87%
The meetings met my expectations for developing new skills	4.90	0.30			10%	90%
Through the meetings I learned about key risk factors for pathological cognitive aging	4.86	0.34			13.3%	86.7%
I apply the general strategies for good cognitive health (healthy diet, exercise, etc.), I learned during the program	4.56	0.62		6.7	30%	63.3%
I learned practical strategies that I can use in my everyday life	4.83	0.42			13.3%	86.7%
The meetings motivated me to take control of my life	4.73	0.44			23.7%	76.3%
The meetings motivated me to take action for my cognitive health	4.76	0.43			23.3%	76.7%
The group format of the intervention was useful	4.80	0.48		3.3%	13.3%	83.3%
I felt supported by the members of my group	4.66	0.59		6.7%	26.7%	66.7%
I felt connected with the members of my group	4.30	0.66		10%	26.3%	63.7%
I routinely apply the memory strategies (e.g., use memory aids, associations, repetition) I learned in the program	4.73	0.52			13.3%	86.7%
I routinely apply the problem-solving steps, I learned in the program (e.g., problem definition, alternative solutions etc)	4.55	0.50		3.3%	20%	76.6%
I routinely apply the emotion regulation strategies (e.g. self-control, diaphragmatic breathing, mindfulness), I learned in the program.	4.86	0.43		3.3%	6.7%	90%
I routinely apply the lifestyle strategies for brain and cognitive health (e.g., physical exercise, diet), I learned in the program	4.33	0.54		3.3%	36.7%	60%

A = very dissatisfied/absolutely disagree/bad, B = dissatisfied/disagree/low, C = neither dissatisfied nor satisfied/neither agree or disagree/moderate, D = satisfied/agree/good, E = very satisfied/strongly agree/very good.

### Applying trained skills

The majority (60–90%) of participants rated their ability to apply their newly learned strategies very highly. Specifically, their ability to apply Memory Compensation Strategies in their daily life was on average 4.73 (SD = 0.52) on a 5-point scale. Their ability to apply Emotion Regulation and Problem-Solving strategies in daily life was evaluated as 4.86 (SD = 0.43) and 4.55(SD = 0.50) respectively. Lastly, their ability to apply general life-style modification strategies to promote overall health (physical exercise and diet) was also rated highly 4.33 (SD = 0.54) on a 5-point scale.

### Association between locus of control and application of trained skills

The internal LC subscale showed a positive correlation with the Memory Compensation Strategies, *r*(28) = 0.48, *p* < 0.001, Problem-Solving, *r*(28) = 0.74, *p* < 0.001, Emotion Regulation, *r*(28) = 0.64, *p* < 0.001 and general modification strategies for brain and cognitive health such as physical exercise and diet, *r*(28) = 0.58, *p* < 0.05. These findings indicate that individuals who reported an internal LC also reported greater engagement in using strategies trained by the program.

## Discussion

The present novel multidimensional group intervention integrates key theoretical principles from cognitive rehabilitation and clinical psychology. This is one of the first multidimensional, non-pharmaceutical, theory driven interventions focusing on healthy adults over 65 with the purpose to strengthen/improve areas of cognitive and psychosocial functioning. The principal objective of the present paper was to present the theoretical framework of this intervention, the process of the program and results from the evaluation that was carried out to investigate participant satisfaction and implementation of their newly learned strategies in their daily lives. The outcomes of this analysis indicate that the intervention is feasible and well tolerated by our target group. Participants were committed to this intervention, as shown by zero dropout.

The intervention incorporated multiple methodologies for contextual integration of learned concepts and strategies: The current intervention adds to existing multidimensional psychoeducational interventions on cognitive aging which were mostly educational (5, 6, 8) through the implementation of multiple methodologies for contextual integration of active components. Specifically, direct instruction through psychoeducation, indirect instruction such as case studies, reflective discussions, interactive instruction such as role-play, peer support, online chatting, and independent study *via* take-home exercises, journals, and case scenarios were integrated during the 10-week period. Results indicate that participants were satisfied with the implementation strategy of the present intervention and with their ability to gain knowledge and apply their newly learned skills.

The intervention incorporated five key active ingredients: Memory Compensation Strategies (MCS), Problem-Solving (PS), Emotion Regulation (ER), Mindfulness, and Locus of Control (LC), in order to address challenges associated with healthy aging. The present findings suggest that, upon completion of the program, participants were able to apply their new knowledge and skills in activities of their daily life, upon completion of the program.

Internal LC, a key ingredient of the present intervention, was associated with participant engagement and their ability to use skills and strategies trained during the program in everyday life. Key aspects of internal locus of control were incorporated in all meetings through active engagement and investment in the learning process. Participants were systematically guided to take action during the group sessions and in their daily lives and to improve their cognitive and psychosocial functioning. Results from the satisfaction questionnaire indicate that most participants reported motivation to take control and action to improve their life. In fact, higher levels of internal LC increased the likelihood of engagement in using strategies trained by the program. This finding has important implications for interventions targeting healthy older adults, since internal LC may maximize the likelihood of active participation in the rehabilitation process by applying compensatory strategies to improve cognition and emotional functioning such as problem-solving and emotion regulation (51).

Another important element of the study was the group format as the delivery modality of the intervention. The group format encouraged the interaction of the participants, elements that potentially contributed to the commitment of the group. The overwhelming majority of participants reported satisfaction with the group format and a connection with their teammates. These results are consistent with a recent meta-analysis showing that interventions produce maximum benefits when participants train in groups (52). The present intervention is conducive to a group format as it offers an opportunity for participants to support each other, increase motivation and allow people to share their emotions and concerns about their cognitive and everyday problems.

Another component of the group intervention in this study was the online delivery method. As mentioned, the online platform was selected as the delivery method in response to the pandemic and the requirement for physical distancing during COVID-19. The present online intervention promoted social interaction during the meetings and the opportunity for online chatting between weekly sessions, creating rapport among the group participants. Findings indicate that the creation of online chats for the group members should be an important consideration when organizing and designing such programs, even if the delivery of the intervention is in the conventional in person format.

In summary, the present study indicates that this theory driven group intervention is feasible and well tolerated by the study participants. Based on the process evaluation and satisfaction results, participants were satisfied with the implementation strategy of the intervention and their ability to gain knowledge and most importantly apply their newly learned skills in their daily life. The current study described the background and content of the intervention program in detail to enable application and replication by other clinicians and researchers. We are currently collecting follow-up data from our study participants and we will be disseminating the outcomes of the intervention to the research community in future publications. In conclusion, the intervention program that was described in the current paper may offer a valuable contribution to the design of a multidimensional intervention and contribution to public health care for older adults, especially in the face of our rapidly aging Western society.

## Data availability statement

The datasets presented in this article are not readily available because the dataset used in this study belongs to an ongoing research project. Therefore, no data can be shared. Requests to access the datasets should be directed to AC, dr.chadjikyprianou@gmail.com and FC, fofic@ucy.ac.cy.

## Ethics statement

The studies involving human participants were reviewed and approved by Cyprus National Bioethics Committee. The patients/participants provided their written informed consent to participate in this study.

## Author contributions

AC conceptualized and designed the project, coordinated the data collection, analyzed data, and wrote the manuscript. FC conceptualized and designed the project, supervised the data collection, analysis, and wrote the manuscript. All authors contributed to the article and approved the submitted version.

## Funding

This work was co-funded by the European Regional Development Fund and the Republic of Cyprus through the Research and Innovation Foundation (EXCELLENCE/1216/0404).

## Conflict of interest

The authors declare that the research was conducted in the absence of any commercial or financial relationships that could be construed as a potential conflict of interest.

## Publisher’s note

All claims expressed in this article are solely those of the authors and do not necessarily represent those of their affiliated organizations, or those of the publisher, the editors and the reviewers. Any product that may be evaluated in this article, or claim that may be made by its manufacturer, is not guaranteed or endorsed by the publisher.
